# Pharyngeal–Cervical–Brachial Variant of Guillain–Barré Syndrome

**DOI:** 10.7759/cureus.18788

**Published:** 2021-10-14

**Authors:** Alberto Moscona-Nissan, Juan Carlos López-Hernández, Alec Seidman-Sorsby, Mayte Cruz-Zermeño, Andrea Navalón-Calzada

**Affiliations:** 1 School of Medicine, Universidad Panamericana, Mexico City, MEX; 2 Neuromuscular Diseases, Instituto Nacional de Neurología y Neurocirugía Manuel Velasco Suárez, Mexico City, MEX

**Keywords:** axonal neuropathy, oropharyngeal and cervicobrachial weakness, inflammatory polyradiculoneuropathy, pharyngeal-cervical-brachial variant, guillain-barré syndrome

## Abstract

Guillain-Barré syndrome (GBS) represents the main cause of flaccid paralysis worldwide. Although most cases have a typical clinical presentation of symmetric ascending flaccid paralysis with areflexia or hyporeflexia, this disease may present as multiple clinical entities, therefore representing a diagnostic challenge for physicians, who should consider these variants when assessing neuropathies. The pharyngeal-cervical-brachial (PCB) variant of Guillain-Barré syndrome is defined by rapidly progressive oropharyngeal and cervicobrachial weakness associated with hyporeflexia or areflexia in the upper limbs. It is present in 3% of all Guillain-Barré syndrome cases and is characterized by axonal rather than demyelinating neuropathy.

We present a pharyngeal-cervical-brachial variant case in a 55-year-old male who presented to the neurological emergency department with a three-day history of progressive and continuous dysarthria, dysphagia to solids, and tongue numbness, later developing paresthesia and weakness in the upper limbs. On physical examination, slight bilateral facial weakness, limited soft palate elevation, absent gag reflex, and limited tongue lateralization were found. Additionally, weakness was found bilaterally in the upper limbs and the flexor and extensor muscles of the neck with preserved muscle strength in the lower limbs. The patient presented upper limb hyporeflexia with lower limb hyperreflexia. A lumbar puncture was performed, revealing protein levels of 35 mg/dL and no cells in the cerebrospinal fluid. Nerve conduction studies reported acute motor and sensory axonal neuropathy (AMSAN). Management of the patient consisted of IgG administration and nasogastric tube insertion.

## Introduction

Guillain-Barré syndrome (GBS) is a disease characterized by acute immune-mediated polyneuropathy. The most common form of GBS is an acute inflammatory demyelinating polyradiculoneuropathy (AIDP), accounting for 85%-80% of total cases [1]. The exact cause and pathophysiology of GBS are not completely understood, although a previous history of an upper respiratory or gastrointestinal tract infection is present in most patients, as well as other infections. The most common infectious agent associated with GBS is *Campylobacter jejuni*. Other infectious agents have been linked to GBS, such as Epstein-Barr virus (EBV), *Mycoplasma*, human immunodeficiency virus, and recently SARS-CoV-2 [1,2]. The typical clinical manifestations of GBS include muscle weakness that normally starts in the legs and ascends to the upper body, hyporeflexia or areflexia when evaluating deep tendon reflexes, and the involvement of cranial nerves [3,4]. Besides AIDP, there are other rare variants of GBS associated with certain antiganglioside antibodies (such as Anti-GQ1b), including Miller Fisher syndrome (MFS) (with classic triad of ataxia, ophthalmoplegia, and areflexia), present in up to 20% of GBS cases, and, less frequently, the pharyngeal-cervical-brachial (PCB) variant, present in 3% of patients with GBS [5-7]. The PCB variant of GBS is rare and represents a diagnostic challenge in emergency services. Cases are reported more frequently in Asian countries, with few reported cases in Latin American countries, such as Mexico.

## Case presentation

A 55-year-old male presented to the neurological emergency department due to a three-day history of progressive and continuous dysarthria and dysphagia to solids accompanied by tongue numbness. Subsequently, the patient presented paresthesia and weakness in the upper extremities. No history of previous illnesses nor a history of infectious disease was present prior to evaluation.

Vital signs were found within normal limits. At the neurological examination, his mental status was normal. Cranial nerve evaluation revealed bilateral facial weakness (incapacity to maintain lid closure against resistance), symmetric uvula with limited soft palate elevation, absent gag reflex, and limited tongue lateralization against resistance. No ophthalmoplegia nor ataxia was reported. When assessing muscle strength, the patient presented weakness in the flexor and extensor muscles of the neck; bilateral and symmetric upper limb weakness with a 2/5 Daniels score in the deltoids, biceps, and hand extensor muscles. Lower limb muscle strength was found with no alterations with a 5/5 Daniels score in the iliopsoas, quadriceps femoris, and tibialis anterior muscles. On reflex examination, the findings showed upper limb hyporeflexia with lower limb hyperreflexia. Plantar reflexes caused a flexor response. Proprioception, exteroception, gait, and cerebellar functions remained unaltered. 

A lumbar puncture was performed on the fourth day of evolution since symptom onset, finding protein levels of 35 mg/dL (normal range: 15-45 mg/dL), glucose levels of 53 mg/dL (normal range: 50-80 mg/dL), and no cells in the cerebrospinal fluid (WBC: 0-5/mm^3^; RBC: 0/mm^3^). Nerve conduction studies reported acute motor and sensory axonal neuropathy (AMSAN). Management of the patient consisted of IgG administration of 2 g/kg divided into five days and nasogastric tube insertion for nutritional support, which was removed four days upon patient admission.

## Discussion

Guillain-Barré syndrome is a condition with an average annual incidence of 0.4-1.7 cases per 100,000 population, affecting predominantly the older population [6]. The prevalence of the pharyngeal-cervical-brachial variant is estimated to be about 3% of all GBS cases, being more prevalent in men than in women (1.3:1), contrary to AIDP GBS, which is more frequently found in the female population. The median age at presentation of the PCB variant is 43 years [7].

Many theories have been proposed in relation to the pathophysiology of GBS and the damage the immune system has on peripheral nerves. Some of these theories include the activation of macrophages with the damage and demyelination of the myelin sheath, the release of toxic mediators, and matrix metalloproteinases, which damage Schwann cells. Moreover, the production of antibodies binding to epitopes on Schwann cells favors complement activation, leading to the destruction of these cells. As previously mentioned, in GBS, an infection is extremely common prior to the presentation of the disease; the introduction of infectious agents, such as *Campylobacter jejuni*, favors a cross-reaction with gangliosides. These mechanisms are known as molecular mimicry-related disease [4]. 

The pathophysiology pathways of GBS have shown that there are antibodies that target specific neuronal gangliosides. In the case of PCB, IgG anti-GT1a and anti-GQ1b antibodies are the most commonly found antibodies. Serological findings can be useful in distinguishing PCB from other GBS variants. The corresponding antibodies in GBS can target different sites in the neuron. For example, in AIDP, antibodies have been shown to target moesin, a protein fundamental in myelination. The typical clinical manifestations of GBS can be understood on the basis of the antibodies that target certain gangliosides in extraocular neuromuscular junctions, dorsal root ganglia, and other structures in the lower part of the brainstem [3,4]. 

The PCB variant of GBS is characterized by a rapid weakness in the oropharyngeal and cervicobrachial area with areflexia in the upper limbs in the absence of ophthalmoplegia or leg weakness. Given that it is an uncommon subtype, its clinical manifestations can be confused with other diseases, such as myasthenia gravis or botulism [7]. In many studies, PCB has been shown to overlap with Miller Fisher syndrome (MFS) [7,8]. The most frequent initial symptoms are proximal weakness of the arm, followed by dysphagia and diplopia. Other less frequent symptoms include blepharoptosis, facial weakness, photophobia, dysgeusia, and psychotic manifestations [9]. Patients who develop ophthalmoparesis and ataxia should be defined as having PCB overlap with MFS, and patients with additional impaired consciousness should be defined as PCB overlap with Bickerstaff brainstem encephalitis (BBE) [8].

The diagnostic criteria for the PCB variant of GBS required for diagnosis are as follows: a) relatively symmetric oropharyngeal weakness, neck weakness, arm weakness, and areflexia/hyporeflexia; b) absence of ataxia and disturbed consciousness and prominent leg weakness; c) monophasic illness pattern and interval between onset and nadir of oropharyngeal or arm weakness between 12 hours and 28 days and subsequent clinical plateau; and d) absence of identified alternative diagnosis [8,10].

The following are the features strongly supportive of the diagnosis: a) previous infectious symptoms; b) cerebrospinal fluid albuminocytological dissociation, with an elevated CSF protein (>45 mg/dL) and a normal CSF white blood cell count (the elevated protein is due to a greater permeability of the blood-brain barrier at the level of the proximal nerve roots; this finding is present in 50%-66% of patients with GBS in the first week after the onset of symptoms and in ≥75% of patients in the third week [8,10]); c) neurophysiological evidence of neuropathy (electrophysiological findings in PCB have demonstrated axonal conduction insufficiency characterized by decreased amplitude of motor (and possibly sensory) responses, with normal conduction velocities [11]); and d) antiganglioside antibodies. In relation to the last feature, a retrospective study of 100 patients found that 70% of patients with PCB have at least one of the following: anti-GT1a IgG antibodies, which were found in 50% of patients; IgG anti-GQ1b antibodies, which were positive in 70% of patients with anti-GT1a antibodies; and IgG antibodies against GM1b, GD1a, or GM1, which were found in 14% of patients. The diagnoses were GBS overlap (11%) or pure PCB (2%). Moreover, anti-GQ1b IgG antibodies (serological markers of MFS and BBE) were positive in 39% of patients with PCB and 30% of patients with pure PCB; these antibodies were more frequently found in patients with PCB overlap with MFS (73%) and BBE (60%). IgG antibodies against GM1, GM1b, GD1a, or GalNAc-GD1a (acute motor axonal neuropathy serological markers) were positive in 27% of patients, being more frequent in GBS overlap (37.5%) than in pure PCB (15%) [12,13].

Patients with GBS can be classified by their clinical (motor-sensory, pure motor, MFS, PCB, etc.) and electrophysiological variants (AIDP, AMSAN, acute motor axonal neuropathy, among others) [7]. In this case, the patient's clinical manifestations correspond to the PCB clinical variant. Nerve conduction studies revealed motor and sensory axonal damage, meeting the criteria for AMSAN electrophysiological variant. 

Considering that there is significant involvement of the oropharyngeal and cervicobrachial muscles in the PCB variant, close monitoring is recommended, and in some cases, nasogastric feeding and ventilator support are necessary. The treatment for GBS, including the PCB variant, consists of symptomatic management and immunotherapy, which includes intravenous immunoglobulin or plasma exchange [1]. In this case, the patient presented a significant improvement of clinical manifestations seven days after IgG administration. Patients with the PCB variant tend to require intubation for secretion management in order to avoid complications such as pneumonia [7]. The prognosis for GBS and the PCB variant varies; most patients recover with minor residual symptoms or signs and may relapse years after. Poor prognosis factors include older age (>50-60), rapid onset before presentation (less than seven days), ventilatory dependency, severely reduced distal compound muscle action potential amplitude (<20% lower limit of normal), preceding CMV infection, diarrheal illness or *Campylobacter jejuni* infection, and Erasmus GBS outcome score at two weeks of >5 [14]. In order to initiate early management and improve patient prognosis, GBS variants should be considered as the cause of diverse clinical presentations, being a diagnostic challenge for physicians. 

## Conclusions

​​Guillain-Barré syndrome represents the main cause of flaccid paralysis worldwide. Although most cases have a typical clinical presentation of symmetric ascending flaccid paralysis with areflexia or hyporeflexia, this disease may present as multiple clinical entities, such as the PCB variant, which is characterized by weakness in the oropharyngeal and cervicobrachial area. When evaluating autoimmune neuropathies, it is fundamental for physicians to consider and suspect GBS variants as the cause of distinct clinical presentations. Further research should be conducted in order to improve clinical diagnosis and management of these Guillain-Barré syndrome variants, such as the PCB variant, and consider it as a differential diagnosis from myasthenia gravis or botulism. 
